# Science periodicals in the nineteenth and twenty-first centuries

**DOI:** 10.1098/rsnr.2016.0026

**Published:** 2016-10-05

**Authors:** Sally Shuttleworth, Berris Charnley

**Affiliations:** CONSCICOM, St Anne's College, University of Oxford, Woodstock Road, Oxford OX2 6HS, UK

**Keywords:** Science, science periodicals, science journals, citizen science, science communication

## The rise of the science periodical in the nineteenth century

From around 100 titles worldwide at the beginning of the nineteenth century, the number of science periodicals grew to an estimated 10 000 by the end, facilitating in the process an exponential growth in popular and professional forms of science.^1^ Nowadays, scientists might take it for granted that publication in a scientific journal is the recognized way to communicate results, but it was only in the early nineteenth century that this practice started to become routine: communication by personal letter or lecture had previously been equally acceptable modes.^2^ Although there has been excellent scholarship on individual aspects of the rise of the scientific journal, more detailed work is needed to understand where the scientific periodical in all its various forms came from, where it might be going and how it has operated over time and in relation to the scientific enterprise.^3^ In this issue we bring together a series of papers on the rise of the science journal, while also placing such developments in the context of the challenges facing contemporary science publishing at a moment when it looks like the twenty-first century science journal, in its physical printed form, might cease to exist.

From scholarship over the last three decades, we have acquired a better understanding of the importance of science periodicals, their diversity and contingent and evolving nature.^4^ This work has been further motivated by the history of science's disciplinary turn to communication.^5^ However, on the detail of how forms and functions varied over time and in relation to other types of communication in print, or oral and epistolary modes, much new ground remains to be broken.^6^ There is a similar shortfall in our knowledge of the developments happening in scientific communication in countries beyond Britain and its immediate partners in scientific exchange, France, Germany and the United States. The international context of correspondence and exchange undoubtedly framed the emergence of both scientific practice and periodical publication. To address some of these issues, we present four detailed studies of the formation and diverse forms of nineteenth-century science periodicals, and their relations to their audiences and commercial contexts, and to other forms of scientific communication.

The history of science periodicals provides a window on to the formation of professional science and many of the scientific practices that have become dominant over the last 200 years. Focusing on the science periodical allows us to pose and explore questions about the construction of science itself. How did the various forms of science periodicals—journals, transactions, proceedings or magazines, to pick just a few of the most obvious examples from a rich and varied culture—help to create and maintain scientific communities and the production of scientific knowledge? Who was involved, and, in the era before the consolidation of professional science, how did elite and non-elite practitioners interact? What was the relationship between, for example, natural history or mechanics' magazines publishing the findings of their readers and the publications of learned societies? And what were the literary forms and devices used to communicate scientific findings to such diverse audiences?

In posing such questions and exploring them through the historical record, we destabilize notions of the scientific periodical as an unproblematic category. Instead we find that the emergence of distinctions between literary and scientific journals, for example, were contingent responses (in this case to the commercial context of publishing, as enterprising publishers sought to open new markets and secure new readers). What is more, the category of ‘scientific’ journal also seems to collapse under the slightest pressure. The shifting forms and functions of the scientific journal were part of a web of interactions between different printed forms—the transactions of learned societies, commercial publications, popular and technical or specialist titles, or indeed encyclopaedias—and the spoken word and performance of science, in popular venues as much as at learned societies.

Such questions (and the provisional answers this special issue provides) are not only of historic interest, but are of crucial significance now, as science has come to utilize new systems of digital and open access publishing, and when questions of expertise and authority are being opened up for scrutiny. If the story of today's science communication is—as is often claimed—one of new technologies facilitating new platforms and new types of communication, this is in fact a very old story. The arrival of the steam-powered press and new paper-making technologies drove the development of Victorian publishing at a furious pace.^7^ Would-be editors in the nineteenth century seized upon and boosted these new technologies with a fervour which shares a great deal with the twentieth and twenty-first centuries' technological developments in publishing. Looking forward, this suggests that there will be no easy or automatic transition from one technology to another, from print to digital or closed to open, but rather a series of choices about what is valuable and worth maintaining.

We are certainly not the first to note that new forms of technology might allow for new forms of communication. This is a dream that runs through the current talk of machine reading and data publication, and the early days of the Internet and digital communication, or Vannevar Bush's hypothetical memex machine and H. G. Wells' *World Brain* in the 1930s and, in the nineteenth century, the Royal Society's *Catalogue of Scientific Papers* (to give just a few examples)*.*^8^ However, despite the obvious extent to which any medium might shape its message, none of these technologies (or putative technologies) was inevitable in its arrival or its action. Even though technologies may contain ‘scripts’—to borrow a term from gender studies—which are formative of the range of possibilities perceived for their use, creators and users played an equally powerful role. The richly peopled histories that follow substantiate the importance of keeping the historical choices made by editors, authors, publishers and readers firmly in view.

The development of what has been termed ‘citizen science’, with the potential online participation of hundreds of thousands of individuals in the creation of scientific knowledge, raises new questions about the boundaries of professional science, and appropriate forms of publication and dissemination. Such questions inevitably lead us back to the nineteenth century when professionalization began to occur. Charles Darwin, with his network of 2000 correspondents, was as happy writing within, and gleaning information from, the pages of the *Gardeners' Chronicle* as any more self-consciously professional scientific journals.^9^ Indeed, ‘his’ correspondents were just as likely to see him as ‘their’ correspondent in their own submissions to the *Chronicle* and a host of titles like it that provided broad authorial access, such as *Science Gossip*, *The Intellectual Observer* or *Knowledge*. Even *Nature*, a direct contemporary and rival to *Knowledge* in its earliest years, aimed to capture the general reader as well as the specialist, advertising itself as designed to satisfy the general ‘laudable craving’ for information on the latest scientific discoveries and ‘the bearing of Science upon civilisation and progress’.^10^ The authorship of *Nature* was, likewise, far from strictly professional in constitution until well into the twentieth century. At a point in time when we are all being encouraged to make our research more open, accessible and relevant, it is instructive to consider the earlier maelstrom of change in the nineteenth century, when commercial journals arose and fell with alarming rapidity as editors and publishers sought to find the most appropriate and appealing forms in which to package science for both general and professional audiences. Indeed, as the articles will make plain, there was no clear division through most of the nineteenth century between professional and popular scientific journals, or between amateur and professional communities.^11^ Journals provided a space both for interaction and for self-definition, as established norms of scientific communication were gradually set in place.

To explore these matters of scientific communication and participation, this special issue brings together papers from leading historians of science and a series of short analysis pieces on contemporary change in scientific publishing from a range of professional perspectives. Both forms of paper are drawn from a symposium jointly hosted by the Constructing Scientific Communities project and the Royal Society on 27 November 2015: ‘The End of the Scientific Journal? Transformations in Publishing, 1800–2015’. Combining historical and contemporary analysis created a productive tension which we aim to replicate in the structure of this volume. While the answers might have altered considerably, we found that questions of form and function, audiences, editorial practices and commercial contexts are as pertinent today as they were in the nineteenth century. Today's debates are part of a growing and evolving set of preoccupations; understanding their roots can help to inform current thinking and decision-making. Moreover, seeing just how unstable the terms of debate have been historically is not only salutatory, it might also liberate those involved in contemporary discussions on the role and future of scientific communication to imagine new forms of expression and scientific practice.

The special issue begins with Jonathan Topham's analysis of four early nineteenth-century scientific journals: Baldwin's *Annals of Philosophy* (1813–1826), Murray and Colburn's *Journal of Science and the Arts* (1816–1830), Constable's *Edinburgh Philosophical Journal* (1819–1864) and Blackwood's *Edinburgh Journal of Science* (1824–1832). Each of these titles, established in the 1810s and 1820s, represented an attempt to mobilize distinctions between the scientific and the literary in order to court popular audiences. The editors and publishers of these titles sought to produce a new type of science journal in what was, ultimately, a failed attempt to capture a new popular audience. These types of alternative, and surprising, configurations in the early history of British science periodical publishing warn strongly against any sort of essentialist reading of the scientific journal's form. They point rather to a rich and diverse history formed as much by the ambitions of publishers and the availability of markets, as by the needs of ‘working men of science’.

In the second contribution, from Pietro Corsi, the troublingly unreliable nature of the scientific journal is brought squarely into focus. Drawing on examples from across continental Europe and beyond, Corsi argues that one should never trust a journal. Dates can be deceitful. Sometimes a scientific publication was not a journal in any sense we would recognize, as when Italian geologists published the *Bollettino del Reale Comitato Geologico d'Italia* (the bulletin of their Geological Survey) in order to draw in foreign periodicals in exchange, before there was very much for them to offer in return. Sometimes quite different forms of publication—encyclopaedias in France, for example—were used in much the same way we might expect journals to be deployed. Once again, we see—this time in the context of an international comparison—that there is no essential form to the scientific journal and that it competed in a packed and varied international market with many other forms of scientific periodical, while itself being suborned in function to the uses of local scientific politics.

From the broad range of practices that might be clustered together under the umbrella of scientific publishing, Bernard Lightman focuses on developments in popular science publishing in the 1860s, 1870s and 1880s. The story is one of transformation as new types of science journal vied for newly literate audiences by promoting a democratizing vision of science. Lightman makes clear that for many journal editors of this period the link between the scientific journal and professional science was not obvious. These were popular science journals that did not merely seek to bring the scientific news of the learned society or the rapidly specializing professional to the general public; instead, they asked their readers to join them in envisioning a radically different ‘Republic of Science’ in which anyone could participate in scientific practice and reap the social, as well as personal, developmental and educational benefits of doing so.

Last in the historical section, Aileen Fyfe and Noah Moxham use two examples of papers that were read to the Royal Society to explore changing practices and gradations of ‘making public’ in the late-eighteenth and nineteenth centuries in another form of scientific periodical: the transactions of learned societies. In charting a broad functional transition from performance at meetings to publication as a key mode of scientific communication, their work throws into sharp relief the challenges to the supposed function of scholarly scientific communication posed by, among other movements, open access. Given the wide range, and sometimes surprising previous functions, of science periodicals, what role do we expect them to play now?

## Looking to the future

The experimentation with forms of scientific publishing apparent in the nineteenth century finds parallels in our era, with the digital revolution apparently opening up possibilities of new forms and modes of publication. Is there, indeed, any need for the scientific periodical in an age of large-scale repositories? And do the calls for open access, and citizen science movements, put under pressure the distinctions which emerged in the twentieth century between professional and public science? We asked our commentators, who are drawn from a range of disciplinary and professional backgrounds, to think broadly about the future of the periodical and science publication. Their provocative and sometimes polemical reflections highlight the roles played by the periodical not only in disseminating scientific results, but also in the wider structures of academic and civic life, from questions of expertise, authority and career incentives, through to issues of patient participation. Whereas the first part of the issue builds a fine-grained analysis of historical developments, this second part, rather like the correspondence sections of a scientific journal, provides a space to explore bold and thought-provoking ideas about what is important in science communication and what should be valued and maintained as we move to new media, new platforms and new forms of engagement.

Vanessa Heggie and Cameron Neylon both address issues of expertise and authority, and the changing role of the scientific journal in relation to the confirmation and demarcation of scientific expertise. Twenty-first century scientific journals, Heggie argues, will continue to sustain scientific authority and expertise, but they will need to have a keener ear for the multiplicity of audiences and modes of reading than the access movement has largely supposed, with its attendant vision of flattened hierarchies. Cameron Neylon, in contrast, looks to expertise and authority as an issue of ‘club goods’ held by disciplines and traded for resources. Journals, he argues, have always played a role in ‘creating groups, defining boundaries and validating membership’; the challenge now is to create new forms of publishing which can maintain these communities while also offering effective engagement with those outside.

Scientific journals have come to play a highly significant and indeed dominating role in current career structures, and both Rebekah Higgitt and Mark Patterson look to the problematic patterns of rewards and incentives thus created. Both suggest looking more creatively at publication forms, with ‘slow scholarship’ or even a reversal of journal growth, so that we return once more to an era of fewer titles. It is, both authors argue powerfully, the very malleability of the past forms and functions of the journal that can help to open up possibilities of thinking differently about how we value, and reward, various modes of publication and dissemination.

Sunetra Gupta and Sabina Leonelli analyse some of the problems and challenges of twenty-first-century scientific publishing. Leonelli focuses on the challenges posed by the move to incorporate data into the publishing process, asking whether dissemination can now extend beyond the formalized, selective and exclusionary writing which is characteristic of much current scientific communication. Gupta argues that the emergence of neoliberalism in the last half-century has pushed the scientific publishing system towards a seemingly more egalitarian, but also more individualistic, emphasis in its operations. The result has been a devaluing of novelty and creativity in science.

Finally, Stella Butler gives the librarian's perspective on recent changes in scientific publishing, while Mary Madden considers the importance of patient and public involvement in research and its dissemination and Raghavendra Gadagkar looks to issues of access to publishing in developing countries. All three examine the implications of open access in these differing contexts. For Stella Butler, the key shift in the library in the last two decades has been a change of function, from the warehousing of print materials to the gatekeeping of electronic resources. One response has been the open access movement, but, Butler warns, that has its limitations. Crucially, the digital environment puts in doubt the continuity and stability of knowledge. Will scholars in the twenty-second century still be able to read the scientific works of the twenty-first century with the same ease with which we can draw upon the printed volumes of the past? Madden's focus is on the unique, but informative, challenges of the British context and of National Health Service research funding. Questions of access, Madden points out, do not stop at the point of publication. For patient and public involvement to be truly democratizing in health care, more conceptual work needs to be done on how information is produced by, and circulates within, patient and public communities. Raghavendra Gadagkar, turning to an international perspective, and especially that of the global south, poses the provocative question: under new pay-to-publish systems, will everyone have the same ability to communicate their research, or will those from less well-endowed institutions be doomed to remain knowledge consumers rather than knowledge producers? Gadagkar believes that access to these systems is uneven, but also inherently unstable; prone to ‘cheating’ by unscrupulous publishers.

As the historical papers in this volume show, the rise of the scientific journal in the nineteenth century was marked by instability and ephemerality, multiplicity and even duplicity, and fierce competition within commercial markets. A history of scientific journals cannot ignore the commercial context and pressures from which the form arose. The rise of the scientific journal was also, however, marked by constant invention and creativity on the part of editors, publishers and contributors as they responded to the pressures of creating and maintaining new audiences and the possibilities opened up by new social contexts and new technologies. Any history of the scientific periodical will inevitably destabilize its own terms of reference; in many ways there is little to hold together the many forms of communication packed into this catchall term. The term is nonetheless a useful one, even as it alerts us to its own instability. The constant tensions between these categories—periodical, journal, transaction, bulletin and so on—show, furthermore, that the history of the scientific journal is not an isolated one; it cannot be told without reference to the many practices—running from the production through to the promotion of a periodical—which held such diverse forms together and in relation to one another and their markets and audiences. The perspective of the past, especially a past when none of the current tenets of science communication were fully established or fixed, offers the possibility of re-imagining science publishing in the future, as our contemporary commentators demonstrate. However, it should also warn us that these are questions of value and maintenance. What do we value about the scientific journal, the transactions or the proceedings and the interdependencies of such forms with scientific practice, and what do we wish to maintain as technologies change? It is our hope that a return to the past will serve to sharpen contemporary debates, alerting us to new (or indeed old) meanings of value, form and function in scientific communication. The history of the science journal is one of perpetual reinvention: although it might indeed cease to exist in paper form, we look forward to tracking its future incarnations.

## Notes

1 See W. H. Brock, ‘Science’, in *Victorian periodicals and Victorian society* (ed. J. Don Vann and R. T. Van Arsdel), pp. 81–96 (University of Toronto Press, 1994), p. 86.

2 J. R. Topham, ‘Anthologizing the book of nature: the circulation of knowledge and the origins of the scientific journal in late Georgian Britain’, in *The circulation of knowledge between Britain, India and China* (ed. Bernard Lightman, Gordon McOuat and Larry Stewart), pp. 119–152 (Brill, Leiden and Boston, 2013), p. 120.

3 See, for example, S. Latham and R. Scholes, ‘The rise of periodical studies’, *PMLA* **121**, 517–531 (2005).

4 See, for example, D. A. Kronick, *A history of scientific and technical periodicals: the origins and development of the scientific and technical press, 1665–1790* (Scarecrow Press, Metuchen, NJ, 1976); A. J. Meadows (ed.), *The development of science publishing in Europe* (Elsevier, Amsterdam, 1980); W. H. Brock, ‘Patronage and publishing: journals of microscopy 1839–1989’, *Journal of Microscopy* **155**, 249–266 (1989); R. Barton, ‘Just before *Nature*: the purposes of science and the purposes of popularization in some English popular science journals of the 1860s’, *Ann. Sci.* **55**, 1–33 (1998); J. A. Secord, *Victorian sensation: the extraordinary publication, reception and secret authorship of* Vestiges of the Natural History of Creation (University of Chicago Press, 2000); J. R. Topham, ‘Scientific publishing and the reading of science in early nineteenth-century Britain: an historiographical survey and guide to sources’, *Stud. Hist. Phil. Sci.* **31**, 559–612 (2000); J. Mussell, *Science, time and space in the late nineteenth-century periodical press* (Ashgate, Aldershot, 2007); N. Hopwood, S. Schaffer and J. Secord, ‘Seriality and scientific objects in the nineteenth century’, *Hist. Sci.* **xlviii**, 251–285 (2010); I. Watts, ‘“We want no authors”: William Nicholson and the contested role of the scientific journal in Britain, 1797–1813’, *Brit. J. Hist. Sci.* **47**, 397–419 (2014); A. Fyfe, J. McDougall-Waters and N. Moxham, ‘350 years of scientific periodicals’, *Notes Rec.* **69**, 227–239 (2015); M. Baldwin, *Making* Nature*: the history of a scientific journal* (University of Chicago Press, 2015).

5 On the turn to communication in history of science, see J. A. Secord, ‘Knowledge in transit’, *Isis* **95**, 654–672 (2004). The publication of the *Wellesley Index to Victorian Periodicals* (1965–1988) offered for the first time an index of article titles, and identifications of anonymous authors for 45 titles of general periodicals, bringing about a real growth in Victorian periodical studies. Science titles, however, were excluded, and history of science has been slower in adopting literary modes of analysis of the publishing forms of science journalism. The ‘Science in the Nineteenth-Century Periodical Project’ (http://www.sciper.org) created a database of references to science in a range of popular periodicals, but there has not been an equivalent project devoted to science periodicals themselves. The recent development of massive digitization of nineteenth-century science periodicals will be a huge boost to scholarship, making titles accessible for the first time to those based outside major libraries. It should be noted, however, that only a small fraction of science journals have so far been digitized, and often limited examples, not full runs.

6 For example, there is, as yet, no thoroughgoing modern history of the *Philosophical Transactions* of the Royal Society. The AHRC project run by Aileen Fyfe, ‘Publishing the *Philosophical Transactions*: the social, cultural and economic history of a learned journal 1665–2015’, is currently producing one. See also A. Fyfe, ‘Journals, learned societies and money: *Philosophical Transactions ca* 1750–1900’, *Notes Rec.* **69**, 3, 277–299 (2015).

7 See A. Fyfe, *Steam-powered knowledge: William Chambers and the business of publishing, 1820–1860* (University of Chicago Press, 2012).

8 V. Bush, ‘As we may think’, *The Atlantic*, July (1945), http://www.theatlantic.com/magazine/archive/1945/07/as-we-may-think/303881/ (accessed 12 July 2016); H. G. Wells, *World brain* (Methuen & Co. Limited, London, 1938); Royal Society, *Catalogue of scientific papers* (C. J. Clay, London, 1867–1925). See also the Royal Society's *International catalogue of scientific literature*, published by subject from the late 1890s. For further analysis of the connections between organization and knowledge, see W. B. Rayward, ‘H. G. Wells's idea of a world brain: a critical reassessment’, *J. Am. Soc. Inf. Sci.* **50**, 557–573 (1999); the now classic J. Beniger, *The control revolution: technological and economic origins of the information society* (Harvard University Press, Cambridge, MA, 1989); and more recently L. Gitelman (ed.), *“Raw Data” is an oxymoron* (MIT Press, Cambridge, MA, 2013). The work of Michael L. Brodie on the ‘Industrial Digital Revolution’ and Neville Brody on the ‘digital public space’ is also highly relevant here, although very little of it appears in standard journal publications. Instead, see http://michaelbrodie.com/ and http://www.rca.ac.uk/more/staff/professor-neville-brody/ respectively (accessed 29 June 2016).

9 See S. Sheets-Pyenson, ‘Darwin's data: his reading of natural history journals, 1837–1842’, *J. Hist. Biol.* **14**, 231–248 (1981).

10 Advertisement for *Nature* in the back pages of B. W. Richardson, *Diseases of modern life* (Macmillan, London, 1876), a work aimed at a popular audience. *Nature*, launched in 1869, was also published by Macmillan.

11 On historical scientific communication structures as a model for modern citizen science, see G. Dawson, C. Lintott and S. Shuttleworth, ‘Constructing scientific communities: citizen science in the nineteenth and twenty-first centuries’, *J. Victorian Cult.* **20**, 246–254 (2015). On popular science in the nineteenth century, see G. Cantor, G Dawson, G. Gooday, R. Noakes, S. Shuttleworth and J. Topham (eds), *Science in the nineteenth-century periodical: reading the magazine of nature* (Cambridge University Press, 2004); L. Henson, G. Cantor, G. Dawson, R. Noakes, S. Shuttleworth and J. R. Topham (eds), *Culture and science in the nineteenth-century media* (Ashgate, Aldershot, 2004); G. Cantor and S. Shuttleworth, *Science serialized: representations of the sciences in nineteenth-century periodicals* (MIT Press, Cambridge, MA, 2004); A. Fyfe, *Science and salvation: evangelicals and popular science publishing in Victorian Britain* (University of Chicago Press, 2004); A. Fyfe and B. Lightman (eds), *Science in the marketplace: nineteenth-century sites and experiences* (University of Chicago Press, 2007); B. Lightman, *Victorian popularizers of science: designing nature for new audiences* (University of Chicago Press, 2007).

